# Effect of a High Protein Weight Loss Diet on Weight, High-Sensitivity C-Reactive Protein, and Cardiovascular Risk among Overweight and Obese Women: A Parallel Clinical Trial

**DOI:** 10.1155/2013/971724

**Published:** 2013-08-06

**Authors:** Leila Azadbakht, Vajihe Izadi, Pamela J. Surkan, Ahmad Esmaillzadeh

**Affiliations:** ^1^Food Security Research Center, Isfahan University of Medical Sciences, Isfahan, Iran; ^2^Department of Community Nutrition, School of Nutrition & Food Science, Isfahan University of Medical Sciences, Isfahan, Iran; ^3^Department of International Health, Johns Hopkins Bloomberg School of Public Health, USA

## Abstract

Studies regarding the effects of high protein (HP) diets on cardiovascular (CVD) risk factors have reported contradictory results. We aimed to determine the effects of an HP diet on CVD risk factors and high-sensitivity C-reactive protein (hs-CRP) among overweight and obese women. In this randomized controlled trial, we recruited 60 overweight and obese women, aged 20–65, into an HP or energy-restricted control diet for three months (protein, carbohydrate, and fat: 25%, 45%, and 30% versus 15%, 55%, and 30%, resp.). Total protein was divided between animal and plant sources in a 1 : 1 ratio, and animal sources were distributed equally between meats and dairy products. Fasting blood samples, hs-CRP, lipid profile, systolic and diastolic blood pressure, and anthropometric measurements were assessed using standard guidelines. Percent change was significantly different between the two diet groups for weight (standard protein (SP): −3.90 ± 0.26 versus HP: −6.10 ± 0.34%; *P* < 0.0001, resp.) and waist circumference (SP: −3.03 ± 0.21 versus HP: −5.06 ± 0.28%; *P* < 0.0001, resp.). Percent change of fasting blood glucose (FBG) substantially decreased in the control group compared to the HP group (−9.13 ± 0.67 versus −4.93 ± 1.4%; *P* = 0.01, resp.). Total cholesterol, systolic blood pressure (SBP), and diastolic blood pressure (DBP) decreased both in the HP and in the control diet groups (*P* = 0.06, *P* = 0.07, and *P* = 0.09, resp.); however, the results were marginally significant. Serum levels of hs-CRP were reduced both in the control (−0.08 ± 0.11%, *P* = 0.06) and in the high protein groups (−0.04 ± 0.09%, *P* = 0.06). The energy-restricted HP diet resulted in more beneficial effects on weight loss and reduction of waist circumference. CVD risk factors may improve with HP diets among overweight and obese women. When using isoenergetic weight loss diets, total cholesterol, hs-CRP, and SBP were marginally significantly reduced, independent of dietary protein content. This trial is registered with ClinicalTrials.gov NCT01763528.

## 1. Introduction

Obesity is a chronic disease that is influenced by an interaction between both genetic and environmental factors [1, 2]. Obesity has emerged as one of the greatest public health problems in the last century [3] and is a leading cause of many other chronic diseases, including hypertension, dyslipidemia, inflammation, type 2 diabetes, cancer, and cardiovascular disease (CVD) [4, 5]. In parallel with the development of obesity, production of adipose tissue derived proteins, such as C-reactive protein (CRP), is usually increased. Elevation of CRP might lead to insulin resistance and CVD [6]. The worldwide prevalence of obesity is rising in developed and developing countries [2], both in adults and in adolescents [1, 7]. Recent research suggests that prevalence of obesity is increasing in Iran, especially in women [5, 8]. 

Moderate weight loss diets, those leading to reduction of 5–10% in body weight, have beneficial effects on CVD risk [9]. According to some investigations, high protein, calorie-restricted diets enhance weight loss, by producing more satiety and reduced energy intake [10, 11] as well as decreased loss of energy expenditure and greater thermogenesis [12, 13]. Many studies have compared the effects of high protein (HP) diets on glycemic control, lipid profiles, and weight loss to other types of calorie-restricted diets [10, 11, 14, 15]. However, results have been contradictory. Some studies have suggested that HP weight loss diets have more capacity to enhance the lipid profile compared with other types of weight loss control groups [14, 16]. In contrast, other studies showed similar results when comparing high protein to normal protein diets [11, 15]. Other research suggests more weight reduction through HP diets both in the short term [15] and in the long term [17]. Several studies have not shown differential effects of diet composition (i.e., protein, carbohydrate, and fat) on weight loss [16, 18]. 

Fewer studies have investigated the effect of HP diets on inflammatory factors like CRP [16, 19]. Additionally, different proteins are likely to have varied effects [16] since consumption of high protein from animal sources, especially red meat, might lead to insulin resistance, bone loss [16, 19, 20], and hypertension [20]. The source of protein is important, and a mixture of animal and plant sources may have enhanced benefits. To our knowledge, recent studies have not evaluated effects of the types of protein present in high protein diets. Furthermore, examining protein intake derived equally from dairy products and meat sources has not been considered in prior studies. Rather, most high protein weight loss diets have focused on animal protein with little attention to vegetable protein intake. Given that few high protein trials have considered the type of protein intake, we examined the effect of an HP weight loss diet composed of 50% plant and 50% animal sources of protein on body weight and cardiovascular risk. 

## 2. Subjects and Methods

### 2.1. Subjects

A convenience sample of sixty-three overweight and obese women, referred to Isfahan Nutrition Clinic, was recruited to participate in this randomized dietary trial between February 2011 and July 2012. Then, simple random sampling was used to randomly allocate subjects into two groups. According to the formula, 17 subjects were needed in each group for adequate power. Subjects were included if they were between 20 and 65 years of age and had a body mass index (BMI or kg/m^2^) of >25, were nonsmokers, and had no history of renal, liver, and metabolic diseases or type 1 or 2 diabetes. Women were excluded if they had gastrointestinal, respiratory, cardiovascular, metabolic, liver, and renal diseases, had macroalbuminuria, or were pregnant or lactating. The study was explained to each subject, after which written informed consent was obtained from all participants. The study was approved by the Research Council and Ethics Committee of the Food Security Research Center, Isfahan University of Medical Science, Isfahan, Iran. This clinical trial is registered with ClinicalTrials.gov as number NCT01763528.

### 2.2. Study Design

Women were randomly assigned to one of the isocaloric energy-restricted diets (a 200–500 kcal reduction of total energy) for three months according to a parallel design while matched on age, BMI, and medication use. Participants were not aware of their dietary group assignment at baseline (i.e., consumption of the high protein diet or standard protein diet). The HP intervention (*n* = 30) was a weight loss diet with 25% of energy from protein, 45% from carbohydrate, and 30% of energy from fat. The control group (*n* = 30) followed a weight loss diet with 15%, 55%, and 30% energy from protein, carbohydrate, and fat, respectively. The total amount of protein was divided between animal and plant sources in a 1 : 1 ratio. Also, animal sources were derived half from meats (e.g., red meat, fish, poultry, egg, and other meat products) and half from dairy products (including milk and yogurt). 

A dietician provided participants with individual regimen consultation and instructions on dietary requirements at the start and once a month throughout the study. No adverse effects were reported by any participant during the study. Participants completed three-day consecutive food records before each visit. Energy and macronutrient intake was analyzed by Nutritionist IV software. A 24-hour physical activity record (in MET) was conducted at the beginning and the end of the study. We used the Maroni formula along with urinary urea nitrogen (UUN), as a marker of protein intake, to assess adherence to the prescribed diets.

### 2.3. Assessment of Anthropometric Measurements and Blood Pressure

Every two weeks, participants were weighed to the nearest 100 grams. Participants were weighed wearing light clothing and without shoes after fasting overnight. At baseline, height was measured using a measuring tape after removal of the participant's shoes. Body mass index (BMI) was calculated by weight (kg)/height (m^2^). At baseline, on a monthly basis and at the end of the study, waist circumference was measured to the nearest 0.1 cm over light clothing at the midpoint between the anterior superior iliac crest and the lower rib at the maximum girth, using nonstretchable tape, without any pressure to the body surface.

After 15 minutes of rest, we measured participants' blood pressure three times in the sitting position and recorded the average of the three measurements. Systolic blood pressure was defined as the appearance of the first sound, and diastolic blood pressure was defined as the disappearance of the sound (Korotkoff phase 5). Blood pressure was measured at week 0 (baseline) and week 12.

### 2.4. Assessment of Biochemical Measurements

Collection of total 24-hour urine output commenced at 07:00 (except for the first morning urine) at weeks 0 and 12. According to standard protocol, fasting blood samples were collected at baseline and week 12 while subjects were sitting. Samples were centrifuged within 30–45 minutes of collection for 10 minutes at 500 ×g and at 40°C. Samples were analyzed using an autoanalyzer (Selectra 2; Vital Scientific, Spankeren, The Netherlands). HDL cholesterol, LDL-c, fasting glucose, and total cholesterol were measured using an enzymatic kit. Triglyceride was measured with glutathione oxidase. High-sensitivity C-reactive protein (hs-CRP) was measured using ELISA and an enzymatic kit. Urinary urea nitrogen (UUN) was determined based on the assessment of protein intake by using the Maroni formula: (protein intake (gr/day) = UUN + 0.031 × weight (kg) × 6.25).

### 2.5. Statistical Analysis

Baseline and end values of cardiovascular risk factors including weight, waist circumference, LDL-c, HDL-c, total cholesterol, fasting blood glucose (FBG), triglyceride (TG), systolic blood pressure (SBP) and diastolic blood pressure (DBP), and hs-CRP in the high protein diet and control diet groups were compared using paired *t*-tests. Percent change in cardiovascular risk factors and hs-CRP in the high protein diet and control diet groups were compared using *t*-tests. Analysis of covariance (ANCOVA) was used to adjust the effects of age, BMI, and medication use on CVD risk. We used SPSS software (version 16.0; SPSS Inc., Chicago IL, USA) for the statistical analyses. *P* < 0.05 was considered statistically significant. 

## 3. Results

**Figure 1:**
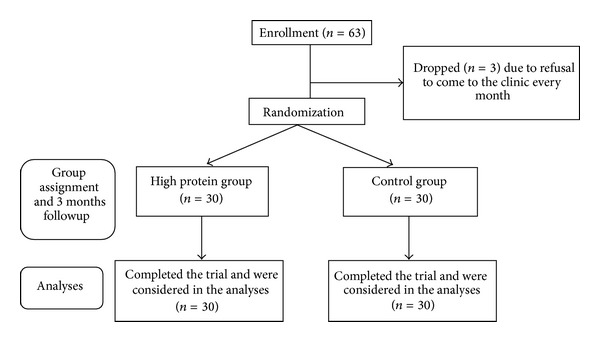
Showing the participants flow diagram of the study.

Of the initial 63 participants in the trial, 3 dropped out due to nonparticipation in the first regimen consultation (*n* = 3). Thus, the study was completed by 60 participants (*n* = 30 subjects for the control group and *n* = 30 subjects for the high protein group) (Figure 1). The mean (±SD) of baseline BMI was 26.8 ± 1.1 versus 27.2 ± 1.2 kg/m^2^ in the control and high protein groups, respectively (*P* = 0.10). The mean age was 40.0 ± 2.4 and 44.1 ± 3.1 years in the control and in high protein groups, respectively (*P* = 0.09). Participant adherence to the diets was assessed by analysis of 24-hour food records. We also assessed compliance to the high protein diet by using the Maroni formula, indicating that participants had relatively good compliance. Medication use among the subjects included Algomed (*n* = 20 in both groups), venustate (*n* = 6 in both groups), SlimQuick (*n* = 22 in both groups), and Dine Bran (*n* = 26 in both groups). Physical activity level was not significant at baseline (21.3 ± 4.6 in control group and 23.0 ± 4.9 MET·h/day in HP group; *P* = 0.34) and at the end of study (26.1 ± 5.5 in control group and 25.2 ± 5.9 MET·h/day in HP group; *P* = 0.44). This was not significantly different even during the study.

Baseline and end values of CVD risk factors and hs-CRP in the high protein and control diet groups are shown in Table 1. Most of the baseline variables were not significantly different except for baseline weight and SBP (*P* = 0.01). Baseline mean (±SD) for weight was 67.93 ± 1.5 kg and 74.13 ± 1.00 kg in the control and high protein groups, respectively (*P* = 0.01). After three months of the intervention, weight, waist circumference, TG, FBG, and SBP were significantly reduced in both the control and high protein diet groups. Total cholesterol was reduced in the control group, which was marginally significant (−6.4 mg/dL, *P* = 0.07). However, this reduction was not significant in the high protein diet group (−2.9 mg/dL, *P* = 0.24). Serum level of LDL-c was substantially changed in the control group (−4.77 mg/dL, *P* = 0.04). However, this parameter was not significantly reduced in high protein group (−2.7 mg/dL, *P* = 0.14). We did not observe any significant change in serum level of HDL-c either in the control or high protein group (*P* = 0.32 and *P* = 0.45, resp.). We observed a marginally significant reduction in DBP in the control group (−4.077 ± 2.3 mg/dL, *P* = 0.05). This reduction was not significant in the high protein group (−1.2 ± 1.8 mg/dL, *P* = 0.31). Serum level of hs-CRP reduced in the control group after three months of the intervention (2.91 ± 1.81 mg/dL at baseline and 2.83 ± 1.75 mg/dL at the end of trial, *P* = 0.05), which was marginally significant. 

Percent changes of cardiovascular risk factors and serum level of hs-CRP in the high protein diet and control groups are presented in Table 2. The percent change in weight and waist circumference was greater in high protein group compared to control group (weight: −6.10 ± 0.34 and −3.9 ± 0.26 kg, *P* = 0.0001 in high protein and control groups, resp.; waist circumference: −5.06 ± 0.28 and −3.03 ± 0.21 cm in high protein and control groups, resp.). Percent change for the serum level of TG, LDL-c, HDL-c, and DBP was not significantly different in the control and high protein groups in crude models or after adjustment for potential confounders. We observed a significant reduction in FBG (*P* = 0.02) and marginally significant reductions in total cholesterol (*P* = 0.09), SBP (*P* = 0.08), and hs-CRP (*P* = 0.07) in the control compared to high protein diet group, even after adjustment for potential confounders. There was no significant difference between high protein diet group and control group in serum levels of HDL-c, after adjustment for potential confounders (0.3 ± 0.42, *P* = 0.68). Serum levels of hs-CRP were reduced in both the control group (−0.08 ± 0.11, *P* = 0.06) and high protein group (−0.04 ± 0.09, *P* = 0.06). 

Forty-one percent and 16% of the patients in HP group lost more than 5% and 10% of their baseline weight, respectively. However, among the controls, 29% and 8% lost more than 5% and 10% of their baseline weight, respectively. 

Table 3 shows the dietary intake of both groups in detail during the study.

## 4. Discussion

Findings of the present study suggest that a high protein low-fat diet had more positive effects on weight and waist circumference reduction compared to a standard high protein diet. However, the control diet conferred more benefits on cardiovascular disease risk factors compared to the high protein diet. To the best of our knowledge, this is the first study to evaluate the type of protein composition and to assess diets with protein sources from all animal types (both low-fat dairy products and different types of meats) as well as plant sources.

According to our study, the energy-restricted HP diet had more beneficial effects on weight loss and waist circumference compared to the control diet. Numerous studies have demonstrated favorable effects of HP diets on weight loss [15, 17, 19, 21]. In this parallel randomized trial among overweight and obese individuals, weight loss in subjects who consumed a high protein low-fat diet (34% protein) was equal to that of subjects who consumed a standard protein high-fat diet (18% protein) [21]. While in another study, an HP meal plan showed more beneficial effects on fat mass reduction among obese adults but found no differences in weight loss between HP and standard protein (SP) groups after 12 weeks [11]. The energy-restricted HP diet (33% protein) combined with resistance exercise for 16 weeks could have a larger impact on weight loss and result in a larger reduction in waist circumference compared with an SP diet (19% protein) among overweight and obese patients with type 2 diabetes [15]. Similar results have been observed in healthy obese individuals [22, 23] and hyperinsulinemic men [24]. Greater weight reduction following an HP energy-restricted diet compared with a high carbohydrate diet may occur due to increasing postprandial thermogenesis [12], as postprandial thermogenesis has been correlated with content of protein in a meal [25, 26]. Protein content in the diet can improve appetite and hunger motivation [27]. A larger protein content in the diet can reduce body weight by mediating more satiety and energy intake reduction [10, 11]. One study demonstrated that consumption of >1.6 gr/kg/day of protein may increase the hypertrophic response to resistance exercise and increase weight reduction maintenance [28]. As obesity, particularly central obesity, is an important risk factor associated with cardiovascular disease and metabolic syndrome [29], an HP weight loss diet is a promising strategy for ameliorating risk factors for CVD. 

Although some prior published research by other investigators has found beneficial effects of high protein diets on lipid profiles [14, 15, 30, 31], we did not observe these results among the overweight and obese women in the present study. Percent change of TG concentration decreased more in the HP diet group compared with the control diet group in our study but was nonsignificant. Previous research has shown that HP diets can improve the lipid profile, independent of weight loss [32]. Another study showed that consumption of an HP diet with 33% protein for 16 weeks reduced TG, TC, and LDL-c but showed no differences compared to the control diet [15]. In our study, percent change in total cholesterol marginally significantly decreased in both the HP and control groups with greater reduction in the control group. Other research has shown that the HP diet has had beneficial effects in reducing LDL-c, TC, and TG and in increasing HDL cholesterol after 64 weeks of weight loss [17]. Also, it has been suggested that the HP content of the diet may result in a greater improvement of TG level because of the diet's lower carbohydrate content [32]. In addition, another study of diabetics reported greater improvement in the lipid profile (TC, TG, LDL-c, and HDL-c) among diabetic patients who adhered to an HP diet [30, 31]. In contrast, others have found that improvement in the lipid profile can occur with weight loss in the absence of dietary protein sources [19]. Noakes et al. showed that markers of CVD risk factors were favorably affected by a weight loss diet with no difference between the HP and control diet groups, except for the TG level which reduced more from the HP diet [16]. Martínez et al. revealed that low content of carbohydrate in the HP diet can lead to reduced VLDL TG production [33]. In contrast to the findings of the present study, the study by Wolfe and Giovannetti reported greater reductions in TC and LDL-c levels [32, 34], and a greater increase in HDL cholesterol [32] has been observed, with no change in TG in response to higher quantities of dietary protein.

 In our study, the baseline of FBG in the HP diet was substantially higher than baseline of FBG in the control group. Percent change of FBG was significantly reduced both in the HP diet and in the control diet groups, with greater reduction in the control group after adjustment for potential confounders. In a parallel trial which was conducted in overweight and obese hyperinsulinemic individuals, the glucose response reduced 6.8% more in subjects who consumed an HP diet (27% protein) compared with the SP group (16% protein) after 16 weeks of a weight loss program [20]. Plasma glucose concentration was significantly improved in both HP and SP groups, with no difference between the two groups [15]. Similar results have been achieved for long-term periods of weight loss among healthy obese women [17]. A significant difference was observed in FBG after 12 weeks of HP meal replacement compared to the SP meal [11].

In our investigation, we were unable to find substantial effects of the HP diet on blood pressure, although marginally significantly reductions in SBP were observed in both diet groups. In another study, HP content had no effect on SBP and DBP in obese hyperinsulinemic patients [20]. In contrast, some previous studies reported lower blood pressure after weight loss, independent of dietary protein content [15, 19]. More research is needed to clarify the impact of HP diets on blood pressure and FBG concentration. 

Our results suggest marginally significantly improvements in hs-CRP among individuals who consumed the HP diet. CRP is a strong predictor of cardiovascular disease which improves following weight loss and reduction of insulin sensitivity [19]. In several studies, CRP concentration has been shown to decrease with weight reduction, independent of dietary composition [16, 17, 19]. Additional research is needed to investigate the influence of HP diets on changes in CRP.

We observed more improvements in CVD risk factors in the control diet compared with the HP diet. Although we distributed the additional protein between plant and animal sources, the animal protein sources were higher in the HP group compared with the control group, such that it may have unfavourably affected some CVD risk factors in the HP group. Although our study suggested that there were improvements in weight among HP groups, the effect of HP diet on other CVD risk factors is not clear. Therefore, more research is needed to more closely evaluate all CVD and other chronic disease outcomes after adherence to an HP diet. 

The present study had several strengths including the fact that we examined the effects of a mixture of animal and plant sources of protein. Animal sources, particularly red meat, may lead to CVD because of their saturated fat [16, 20]. Also, we were interested in low fat dairy products as animal sources, which may lead to attenuation of bone loss [35], an important concern in postmenopausal women. We controlled some important potential confounders which may affect CVD risk factors. Additionally, use of the Maroni formula in conjunction with the analysis of dietary intake aided in assessing protein intake. One limitation is that we were not capable of blinding the dietitian because we were using a dietary intervention. Also, as the trial was conducted among only women, we cannot generalize the results to the general population. The study follow-up period was relatively short, only three months. Given the varied effects of dietary interventions depending on the intervention duration, additional studies with longer follow-up periods are needed. Longitudinal dietary interventions are important in order to gain a better understanding of long-term diet adherence and more precise estimates of the effects. However, difficulties such as budget limitations and lower compliance of participants in longitudinal dietary trials may provide challenges. To further assess the importance of HP diets on CVD risk factors, further research should be conducted using varied proportions of protein, and research should be conducted in different populations with longer intervention periods. Participants did not mention any specific adverse events in the present study. This might be due to the kinds of protein sources in our prescribed diet compared to those of previous studies, as we provided a high protein diet using varied sources of protein. One kind of dairy that is often preferred by Iranians is yogurt, which may also protect from many gastrointestinal disorders. These balanced sources of dietary protein may be one reason that we did not receive any reports of adverse effects. As the reports show some unfavorable dietary behaviors among Iranian population [36, 37], conducting interventional dietary research to clarify the suitable diet is necessary.

## 5. Conclusion

Both prescribed diets had positive effects on anthropometric measurements, but the HP diet resulted in a greater reduction of body weight and waist circumference. Under isoenergetic weight loss diets, total cholesterol, hs-CRP, and SBP were marginally significantly reduced independent of dietary protein content. We were unable to observe significant changes in DBP, HDL-c, and LDL-c cholesterol in the present study. FBG was substantially reduced in both diet groups with a greater reduction in the control group. Hence, an HP diet consisting of 50% plant and 50% animal sources of protein can reduce weight and waist circumference more than a standard protein diet among overweight and obese women. Further investigations are warranted to confirm these findings and elucidate the potential mechanisms that may explain the changes in anthropometric measurements following an HP diet.

## Figures and Tables

**Table 1:** Baseline and end values of cardiovascular risk factors and hs-CRP in the high protein diet and control diet groups^1^.

Variables	Control group (*n* = 30)	High protein diet group (*n* = 30)	*P* value^2^
Weight (kg)			
Baseline	67.93 ± 1.5	74.13 ± 1.00	0.01
End	64.00 ± 1.5	68.03 ± 1.10	0.03
*P* ^3^	0.01	0.01	—
BMI (kg/m^2^)			
Baseline	26.8 ± 1.1	27.2 ± 1.2	0.09
End	26.5 ± 1.0	26.7 ± 1.1	0.29
*P* ^3^	0.10	0.07	
Waist (cm)			
Baseline	103.97 ± 1.2	102.27 ± 1.6	0.41
End	100.93 ± 1.2	97.20 ± 1.5	0.06
*P* ^3^	0.09	0.05	—
Triglyceride (mg/dL)			
Baseline	177.03 ± 6.9	179.47 ± 6.9	0.80
End	170.70 ± 6.9	172.40 ± 7.1	0.86
*P* ^3^	0.04	0.03	—
FBG (mg/dL)			
Baseline	112.93 ± 3.6	119.50 ± 3.1	0.20
End	103.80 ± 3.7	114.57 ± 3.1	0.03
*P* ^3^	0.03	0.07	—
Total cholesterol (mg/dL)			
Baseline	214.87 ± 6.4	212.90 ± 5.2	0.81
End	208.47 ± 6.4	210.00 ± 5.2	0.85
*P* ^3^	0.07	0.24	—
LDL cholesterol (mg/dL)			
Baseline	113.20 ± 3.3	117.30 ± 3.6	0.41
End	108.43 ± 3.5	114.60 ± 3.2	0.20
*P* ^3^	0.04	0.14	—
HDL cholesterol (mg/dL)			
Baseline	42.20 ± 1.6	43.33 ± 1.6	0.54
End	42.20 ± 1.04	43.63 ± 1.6	0.46
*P* ^3^	0.32	0.45	—
Systolic blood pressure (mmHg)			
Baseline	137.67 ± 3.1	127.73 ± 2.2	0.01
End	132.40 ± 2.8	124.07 ± 2.3	0.03
*P* ^3^	0.08	0.09	—
Diastolic blood pressure (mmHg)			
Baseline	81.83 ± 2.3	81.50 ± 1.8	0.91
End	77.76 ± 2.5	79.30 ± 1.9	0.63
*P* ^3^	0.05	0.31	—
hs-CRP (mg/dL)			
Baseline	2.91 ± 1.81	2.88 ± 1.76	0.77
End	2.83 ± 1.75	2.84 ± 1.77	0.54
*P* ^3^	0.05	0.18	—

^1^Values are mean ± SEM.

^2^These *P* values resulted from *t*-tests between the baseline values and also *t*-tests between the end values separately; *P* < 0.05 was considered statistically significant.

^3^These *P* values resulted from paired *t*-test between baseline and end values in each group individually; *P* < 0.05 was considered statistically significant.

**Table 2:** Percent changes of cardiovascular risk factors and hs-CRP in high protein diet and control diet groups after adjustment for age, BMI, and medication use^1^.

Variables	Control diet group	HP diet group	*P* value^2^
Weight	−3.90 ± 0.26	−6.10 ± 0.34	0.0001
Waist	−3.03 ± 0.21	−5.06 ± 0.28	0.0001
Triglyceride	−6.33 ± 1.9	−7.06 ± 1.4	0.76
Triglyceride adjusted	−6.21 ± 1.6	−6.37 ± 1.3	0.83
Fasting blood glucose	−9.13 ± 0.67	−4.93 ± 1.4	0.01
Fasting blood glucose adjusted	−8.67 ± 0.56	−4.32 ± 1.3	0.02
Total cholesterol	−6.4 ± 1.3	−2.9 ± 1.2	0.06
Total cholesterol adjusted	−6.1 ± 1.0	−2.7 ± 1.0	0.09
LDL cholesterol	−4.76 ± 1.05	−2.7 ± 0.20	0.20
LDL adjusted	−4.64 ± 0.97	−2.6 ± 0.10	0.24
HDL cholesterol	0.0001 ± 0.5	0.3 ± 0.47	0.66
HDL adjusted	0.0001 ± 0.5	0.3 ± 0.42	0.68
Systolic blood pressure	−5.26 ± 0.73	−3.66 ± 0.47	0.07
Systolic blood pressure adjusted	−5.06 ± 0.67	−3.69 ± 0.41	0.08
Diastolic blood pressure	−4.06 ± 0.84	−2.2 ± 0.68	0.09
Diastolic blood pressure adjusted	−4.01 ± 0.78	−2.11 ± 0.65	0.10
Hs-CRP	−0.08 ± 0.11	−0.04 ± 0.09	0.06
Hs-CRP adjusted	−0.07 ± 0.10	−0.03 ± 0.09	0.07
BMI	−0.39 ± 0.02	−0.57 ± 0.03	0.01

^1^Values are mean ± SEM.

^2^These *P* values resulted from *t*-tests between the control diet group and the high protein diet group; *P* < 0.05 was considered statistically significant.

**Table 3:** Mean ± SE of energy and nutrients intake of control diet and high protein diet during the study^1^.

Variables	Control group	High protein group	*P* values^2^
Energy (kcal/day)	2060 ± 731	2099 ± 796	0.21
Carbohydrate (% of total energy)	56.2 ± 3.7	45.7 ± 1.5	0.01
Protein (% of total energy)	14.7 ± 1	23.9 ± 2.7	0.01
Fat (% of total energy)	29.1 ± 3.2	31.4 ± 2.9	0.31
Fiber (gr/day)	20.1 ± 7.1	17.6 ± 7	0.17
Cholesterol (gr/day)	256 ± 39	293 ± 41	0.19
Whole grain (gr/day)	25.0 ± 0.11	20.1 ± 10.5	0.09
Refine grain (gr/day)	219 ± 71	182 ± 61	0.05
Fish (gr/day)	10.3 ± 3.3	8.0 ± 3.1	0.37
Poultry (gr/day)	26.1 ± 3.9	49.9 ± 6.1	0.01
Fruits (gr/day)	193 ± 66	153 ± 61	0.09
Vegetables (gr/day)	171 ± 51	153 ± 61	0.14
Nuts (gr/day)	5.2 ± 1.3	9.9 ± 2.4	0.01
Meats (gr/day)	23.1 ± 4.9	45.1 ± 5.6	0.01
High fat dairy products (gr/day)	257 ± 69	353 ± 123	0.01
Low fat dairy products (gr/day)	60 ± 45	120 ± 33	0.01
Legumes (gr/day)	63 ± 21	93 ± 39	0.12

^1^Values are mean ± SEM.

^2^These *P* values resulted from *t*-tests between the control diet group and the high protein diet group; *P* < 0.05 was considered statistically significant.
